# Carbon–Cellulose Hybrid Materials for Microplastics Removal: Adsorption Mechanisms, Structure–Function Relationships, and Current Challenges

**DOI:** 10.3390/nano16120710

**Published:** 2026-06-09

**Authors:** Rabiga M. Kudaibergenova, Aitekova R. Anar, Seitzhan A. Orynbayev

**Affiliations:** Department of Chemistry and Chemical Technology, Faculty of Technology, M. Kh. Dulaty Taraz University, Taraz 080000, Kazakhstan; orynbayev2050@gmail.com

**Keywords:** microplastics, carbon–cellulose nanocomposites, nanomaterials, adsorption, porous structures, hybrid materials, wastewater treatment, environmental remediation, sustainable development goals (SDGs), SDG 6

## Abstract

Microplastics (MPs, plastic particles < 5 mm) and nanoplastics (NPs, plastic particles generally <1 µm), collectively referred to as micro/nanoplastics (MNPs), have emerged as critical contaminants in wastewater systems due to their persistence, small size, and ability to act as vectors for co-contaminants. Conventional wastewater treatment technologies are often insufficient for the effective removal of microplastics, particularly for smaller particles and nanoplastics, necessitating the development of functional materials and innovative treatment strategies. In this review, recent advances in carbon-based materials, cellulose-based materials, and their hybrid carbon–cellulose composites for microplastics removal are critically analyzed and comparatively discussed. Particular attention is given to the structure–function relationships governing adsorption performance, including the roles of hierarchical porosity, surface chemistry, and interfacial interactions. The key mechanisms responsible for microplastics capture—such as hydrophobic interactions, π–π stacking, hydrogen bonding, electrostatic attraction, physical entrapment, and pore trapping—are systematically discussed. Carbon–cellulose composite materials are highlighted as a promising class of multifunctional adsorbents due to their synergistic combination of hydrophilic cellulose scaffolds and hydrophobic carbon domains. This dual functionality enables efficient removal of microplastics across a wide range of sizes and morphologies. Recent developments in magnetic and superhydrophobic composite systems further demonstrate enhanced separation efficiency, recyclability, and potential applicability in real wastewater environments. In addition to summarizing recent progress, this review critically examines the methodological inconsistencies, mechanistic uncertainties, and practical limitations associated with current adsorption systems. Despite significant progress, several challenges remain, including the lack of standardized evaluation methods, limited validation under real wastewater conditions, material stability issues, and scalability constraints. Future research directions are proposed, focusing on rational material design, sustainable carbon sources, multifunctional hybrid systems, and integration into existing treatment infrastructures. The development of sustainable hybrid adsorption systems for microplastics remediation also contributes to the achievement of Sustainable Development Goal 6 (Clean Water and Sanitation) by supporting improved wastewater treatment technologies and reduction in emerging aquatic contaminants.

## 1. Introduction

Microplastic (MP) pollution has emerged as a major environmental challenge among emerging pollutants due to the persistence, ubiquity, and complex environmental behavior of plastic particles smaller than 5 mm [1,2]. Their small size, high surface area, and ability to act as vectors for toxic pollutants and microorganisms make microplastics particularly problematic contaminants in aquatic systems. Wastewater treatment plants (WWTPs) are recognized both as barriers and secondary pathways for microplastics, as conventional treatment processes do not ensure complete removal, particularly for smaller particles, fibers, and nanoplastic fractions [2,3]. The occurrence of polyethylene (PE), polypropylene (PP), polystyrene (PS), and polyethylene terephthalate (PET) microplastics in municipal and industrial wastewater has raised increasing concern due to their potential ecological risks, capacity to adsorb co-contaminants, and possible transfer through aquatic food webs [1,2,3]. In addition, environmental aging, oxidation, and biofilm formation may substantially alter the surface chemistry, transport behavior, and adsorption properties of microplastics in wastewater systems, further complicating their removal [2,3].

Existing approaches for microplastics removal include membrane filtration, coagulation–flocculation, flotation, advanced oxidation, and adsorption [2,4]. Among these, adsorption has attracted increasing attention because of its operational simplicity, scalability, and effectiveness toward heterogeneous and irregularly shaped microplastic particles [4]. However, the development of sustainable, regenerable, and high-performance adsorbents remains a critical challenge. Moreover, reported removal efficiencies often vary substantially depending on polymer type, particle size, water chemistry, and operational conditions, making direct comparison between treatment systems difficult [2,4].

Cellulose-based materials, including cellulose nanofibers (CNFs), cellulose nanocrystals (CNCs), bacterial cellulose, and porous cellulose aerogels, have emerged as promising sustainable adsorbents owing to their renewability, tunable surface chemistry, low density, hierarchical porosity, and abundance of hydroxyl groups available for functionalization [5,6,7]. Recent studies have demonstrated that nanocellulose-derived materials can contribute to microplastic capture through physical entrapment, interfacial interactions, and surface adsorption mechanisms [1,5]. However, pristine cellulose exhibits intrinsic limitations, particularly its hydrophilic nature, which reduces affinity toward hydrophobic polymer particles such as PE and PP. In addition, the relative contribution of adsorption, pore trapping, and physical filtration mechanisms in cellulose-based systems remains insufficiently clarified in many studies.

Carbon-based materials, including graphene oxide (GO) [8,9], carbon nanotubes (CNTs) [10], activated carbon [11], and biochar [12], are widely recognized for their large specific surface area, surface reactivity, hydrophobic domains, and strong adsorption potential [13,14,15]. In the context of microplastics removal, carbon materials may contribute through hydrophobic interactions, van der Waals attraction, π–π interactions (particularly for aromatic polymers such as PS), and porous entrapment mechanisms [4,13,15]. Nevertheless, challenges related to aggregation, recovery, and structural stability may limit the direct application of individual carbon nanomaterials in aqueous systems. Furthermore, the adsorption performance of carbon-based materials is strongly influenced by pore structure, surface oxidation degree, particle dispersion, and environmental conditions, resulting in considerable variability among reported studies.

Combining carbon materials with cellulose into hybrid carbon–cellulose composite materials offers a promising strategy to generate multifunctional adsorbents that integrate the structural advantages of cellulose with the interfacial and adsorptive functionality of carbon materials. Such composites exhibit synergistic effects between cellulose scaffolds and carbon nanostructures, resulting in enhanced surface area, improved mechanical integrity, tailored wettability, interconnected porous architectures, and improved reusability [6,13,16]. Particularly, porous carbon–cellulose aerogels, cellulose–GO networks, cellulose–CNT sponges, and cellulose–biochar composites have attracted growing interest in water purification applications. At the same time, important challenges related to scalability, regeneration, nanomaterial stability, and realistic wastewater performance remain insufficiently addressed.

Although previous reviews have discussed microplastics remediation technologies, nanocellulose materials, and carbon-based adsorbents separately, a focused critical discussion of carbon–cellulose hybrid systems for microplastics removal remains comparatively limited. Existing reviews have largely examined microplastics removal in general [2,4], nanocellulose-based materials [1,5], or cellulose/carbon composites for broader water treatment applications [16], without critically integrating adsorption mechanisms, structure–function relationships, and practical limitations specifically associated with hybrid carbon–cellulose systems for microplastics remediation.

Therefore, this review provides a focused critical synthesis of recent advances in carbon–cellulose composite materials for microplastics removal from wastewater. Particular emphasis is placed on (i) the characteristics and challenges associated with microplastics removal, (ii) the design and classification of carbon–cellulose composites, (iii) the underlying adsorption and capture mechanisms, (iv) performance evaluation and reusability, and (v) current limitations and future research directions. This review critically analyzes current progress, mechanistic complexities, methodological inconsistencies, and practical challenges associated with carbon–cellulose hybrid adsorbents for realistic wastewater treatment applications.

## 2. Literature Search Methodology

This review was conducted as a narrative critical review aimed at summarizing and critically analyzing recent advances in carbon–cellulose composite materials for microplastics removal from wastewater systems [17,18]. The literature survey focused primarily on peer-reviewed journal articles addressing adsorption mechanisms, material design, physicochemical properties, and practical challenges associated with carbon-based, cellulose-based, and hybrid composite adsorbents.

Scientific publications were collected from major academic databases, including Scopus, Web of Science, ScienceDirect, and Google Scholar. The literature search was primarily focused on studies published between 2015 and 2025 in order to capture recent developments in microplastics remediation technologies and advanced hybrid adsorbent materials [19,20].

The search strategy included combinations of keywords such as “microplastics removal”, “microplastics adsorption”, “carbon–cellulose composites”, “nanocellulose adsorbents”, “graphene oxide microplastics”, “carbon nanotubes wastewater treatment”, “biochar composites”, “hybrid adsorbents”, and “microplastics wastewater treatment”. Additional relevant references were identified through cross-referencing of selected review papers and research articles [1,4].

The inclusion criteria considered studies published in English that focused on the removal, adsorption, filtration, or capture of microplastics and nanoplastics in aqueous systems using cellulose-based materials, carbon-based adsorbents, or hybrid composite structures. Studies unrelated to wastewater treatment, non-aqueous systems, or conventional macroplastic remediation were excluded from detailed analysis.

The collected literature was screened based on title, abstract, and full-text relevance. Particular attention was given to studies reporting material synthesis strategies, adsorption mechanisms, removal efficiency, structural characterization, operational stability, regeneration behavior, and performance under realistic wastewater conditions.

Approximately 150–180 peer-reviewed publications were initially screened during the literature survey. Following relevance evaluation and exclusion of studies not directly related to wastewater-based microplastics remediation using cellulose-, carbon-, or carbon–cellulose-based materials, approximately 90–110 highly relevant studies were critically analyzed and comparatively discussed in this review.

Rather than providing a fully systematic or bibliometric survey, this review aims to present a focused critical synthesis of current progress, mechanistic understanding, and practical challenges associated with carbon–cellulose hybrid materials for microplastics remediation.

## 3. Microplastics in Wastewater: Characteristics and Challenges

Microplastics (MPs) in aquatic environments originate from both primary sources, such as industrial pellets, cosmetic microbeads, and synthetic fibers, and from secondary fragmentation of larger plastic debris resulting from weathering and mechanical degradation processes [19,20,21]. Wastewater treatment plants (WWTPs) act as major interception points for microplastics; however, they also function as secondary emission sources due to incomplete removal efficiency, particularly for smaller particles, fibers, and nanoplastic fractions [20,21,22,23].

The physicochemical properties of microplastics strongly influence their behavior in wastewater systems. These particles typically consist of polymers such as polyethylene (PE), polypropylene (PP), polystyrene (PS), and polyethylene terephthalate (PET), which exhibit varying densities, crystallinity, and surface hydrophobicity [19,21,22]. Consequently, microplastics may float, remain suspended, or settle depending on their intrinsic properties and environmental conditions. Their small size and high surface-area-to-volume ratio enhance their ability to adsorb organic pollutants, heavy metals, pharmaceuticals, and microorganisms, thereby increasing their ecological risk [19,22,23]. However, the extent to which adsorbed contaminants contribute to the overall toxicity and transport behavior of microplastics remains actively debated, particularly under dynamic wastewater conditions. Furthermore, environmental aging and biofilm formation (plastisphere development) can significantly alter their surface chemistry, transport behavior, and reactivity during wastewater treatment processes [22,23,24].

One of the main challenges in microplastics removal is their wide size distribution, ranging from several millimeters to nanoplastics (<1 µm), which limits the effectiveness of conventional treatment technologies [20,21,25]. Primary treatment processes such as screening and sedimentation are generally more effective for larger particles, whereas secondary biological treatment may not efficiently capture smaller and buoyant microplastics [4,18]. Even advanced tertiary treatments, including membrane filtration, coagulation–flocculation, and advanced oxidation processes, may suffer from membrane fouling, high operational costs, or incomplete removal of nanoscale particles [23,25]. In addition, reported removal efficiencies for advanced treatment technologies vary substantially among studies due to differences in particle size distribution, analytical methodology, and operational conditions.

Another major challenge is the diverse morphology of microplastics, including fibers, fragments, spheres, and films, which significantly affects transport behavior, aggregation dynamics, and interactions with adsorbent materials [3,24,26]. Fibrous microplastics, in particular, are difficult to remove due to their high aspect ratio and tendency to bypass conventional filtration systems [3,26]. Their elongated morphology and flexibility may facilitate bypass through filtration systems while simultaneously promoting entanglement and fouling within treatment units.

Reported removal efficiencies in WWTPs vary substantially depending on treatment configuration, hydraulic conditions, influent composition, and sludge management practices [19,24,25]. Importantly, smaller microplastics and nanoplastics may pass through treatment barriers and enter receiving water bodies, representing a critical unresolved environmental challenge [24,25,27,28].

Another significant limitation is the lack of standardized analytical methods for microplastics detection and quantification, particularly for smaller fractions and nanoplastics. As a result, direct comparison of microplastics concentrations and treatment efficiencies reported in the literature remains highly challenging, particularly for nanoplastics and weathered particles. This complicates cross-study comparisons and hinders the reliable evaluation of emerging treatment technologies [27,28].

Therefore, the development of advanced functional materials capable of effectively capturing microplastics across a wide range of sizes, morphologies, and surface chemistries is urgently required. This has driven increasing interest in porous, functionalized, and hybrid adsorbents, including cellulose-based and carbon-based composite materials [1,20,22,29].

## 4. Cellulose-Based Materials for Microplastics Removal

Cellulose-based materials have attracted increasing attention in environmental remediation due to their biodegradability, natural abundance, low toxicity, and highly tunable surface chemistry [1,30,31,32]. As the most abundant biopolymer on Earth, cellulose exists in multiple structural forms, including microcrystalline cellulose, nanocellulose—such as cellulose nanofibers (CNFs) and cellulose nanocrystals (CNCs)—and bacterial cellulose, each offering distinct physicochemical properties relevant to water treatment applications [2,32].

Cellulose-based scaffolds include not only nanocellulose, but also cellulose fibers, woven and non-woven mats, membranes, filter papers, sponges, and aerogels. These materials differ in fiber diameter, porosity, water uptake, surface charge, and mechanical stability, which directly influence microplastic capture and interfacial bonding in composite matrices.

Nanocellulose can be produced through mechanical fibrillation, chemical pretreatment-assisted disintegration, or bacterial biosynthesis. Mechanical methods mainly reduce fiber dimensions and increase fibrillation, whereas chemical approaches such as oxidation can introduce charged functional groups and improve dispersion stability. Bacterial cellulose forms highly pure nanofibrillar hydrogel networks with distinct morphology and water retention capacity. These production routes strongly influence surface charge, fiber morphology, and compatibility with carbon nanomaterials.

Among these, nanocellulose has emerged as a particularly promising platform for microplastics removal due to its high specific surface area, abundant hydroxyl functional groups, and ability to form interconnected three-dimensional porous networks [31,32,33]. Cellulose is also hygroscopic and capable of absorbing water, which is important for swelling behavior, wettability, and stability in aqueous treatment systems. These features facilitate multiple interaction pathways with microplastic particles, including hydrogen bonding, physical entrapment, and interfacial adhesion. Surface modification and chemical functionalization have further been shown to improve interaction with hydrophobic polymer particles and enhance adsorption performance [4,31,33].

However, despite the generally promising adsorption performance reported for nanocellulose-based systems, the dominant removal mechanisms remain incompletely understood. Some studies primarily attribute microplastics capture to hydrogen bonding and surface adsorption, whereas others emphasize the importance of pore confinement and physical entrapment within hierarchical porous structures [31,32,33]. This inconsistency complicates the interpretation of structure–performance relationships and indicates that adsorption mechanisms may strongly depend on pore architecture, polymer type, and surface chemistry.

Bacterial cellulose has also attracted considerable attention due to its highly pure nanofibrillar structure, high crystallinity, mechanical strength, and water retention capacity, making it suitable for reusable water treatment systems [34]. In parallel, cellulose aerogels have gained increasing interest because of their ultralow density, interconnected porous structure, and high adsorption capacity toward fibrous and irregularly shaped microplastics [33]. Their hierarchical pore networks facilitate efficient particle transport and mechanical trapping across a broad size range.

Nevertheless, adsorption efficiencies reported for cellulose aerogels and nanocellulose membranes vary substantially across the literature. These discrepancies are largely associated with variations in pore size distribution, polymer composition, particle size, and experimental conditions. In many cases, adsorption studies are performed using simplified synthetic suspensions rather than real wastewater matrices, which may lead to overestimation of removal efficiency under practical conditions [4,30]. In addition, the lack of standardized methodologies for microplastics quantification and adsorption evaluation complicates direct comparison between reported systems.

Despite these advantages, pristine cellulose materials exhibit important limitations. Their hydrophilic nature reduces affinity toward hydrophobic microplastics such as polyethylene (PE) and polypropylene (PP), limiting adsorption efficiency in complex wastewater systems containing diverse organic contaminants [13,30,35]. Furthermore, swelling behavior and partial deformation of porous cellulose structures during prolonged aqueous exposure may reduce pore accessibility and compromise long-term operational stability. Fouling caused by dissolved organic matter and biofilm accumulation may additionally block active adsorption sites and decrease transport efficiency within porous networks.

To address these limitations, various surface engineering and oxidation strategies have been developed, including TEMPO-mediated oxidation, esterification, carboxylation, and grafting of hydrophobic functional groups [4,33,35]. In particular, TEMPO-mediated oxidation is widely applied not only for surface functionalization, but also as a pretreatment approach for nanocellulose production through selective oxidation of cellulose hydroxyl groups. These approaches improve surface reactivity, wettability control, and interaction with hydrophobic polymer particles. However, excessive oxidation or aggressive chemical modification may alter pore morphology and reduce structural integrity. In addition, real wastewater matrices containing dissolved organic matter, salts, surfactants, suspended solids, and competing pollutants may further increase competitive adsorption effects. Such trade-offs remain insufficiently investigated in current studies.

Recent advances have also focused on the fabrication of nanocellulose-based membranes, sponges, and hierarchical porous architectures with improved mass transfer properties and enhanced adsorption efficiency [31,35]. In addition, cellulose has been integrated with graphene oxide, carbon nanotubes, activated carbon, and biochar to construct multifunctional hybrid composites with improved mechanical stability and interfacial adsorption properties.

These hybrid systems benefit from synergistic interactions between cellulose scaffolds and carbon nanostructures, enabling the coexistence of hydrophilic transport pathways and hydrophobic adsorption-active domains. Nevertheless, the actual contribution of these combined effects remains difficult to quantify due to significant methodological inconsistencies among studies, including variations in carbon loading, pore structure, surface chemistry, and adsorption conditions [36]. In several cases, improved performance has been attributed to carbon incorporation without clearly distinguishing between contributions from surface adsorption, hydrophobic interactions, and physical filtration effects. Therefore, more rigorous structure–function investigations are required to establish reliable design principles for high-performance cellulose-based hybrid adsorbents.

Overall, cellulose-based materials represent a sustainable and versatile platform for microplastics removal. However, their adsorption performance strongly depends on surface modification, pore architecture, and operational conditions. The integration of cellulose with functional carbon-based materials offers a promising pathway toward the development of multifunctional hybrid adsorbents with improved efficiency, selectivity, and long-term stability.

## 5. Carbon-Based Materials for Microplastics Removal from Wastewater

**Figure 1:**
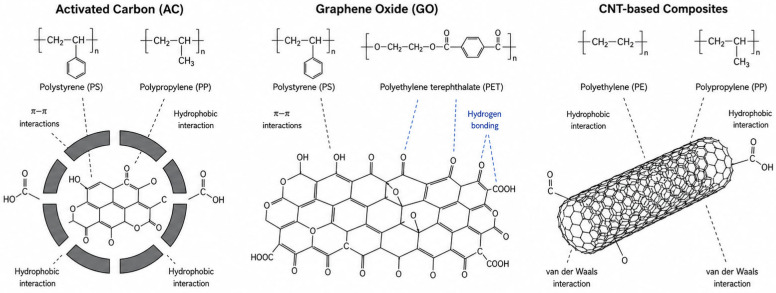
Schematic illustration of dominant adsorption mechanisms between microplastic polymers and carbon-based adsorbents, including hydrophobic interaction, π–π stacking, and pore trapping.

Carbon-based materials have been widely investigated as advanced adsorbents for environmental remediation due to their high specific surface area, tunable surface chemistry, structural diversity, and strong affinity toward organic and particulate contaminants [37,38,39]. In the context of microplastics removal, materials such as graphene oxide (GO), carbon nanotubes (CNTs), activated carbon, and biochar have demonstrated significant potential owing to their ability to interact with polymeric particles through multiple interfacial mechanisms. The main interaction pathways between microplastics and carbon-based materials are schematically summarized in Figure 1. The adsorption of microplastics onto carbon-based materials is primarily governed by three dominant interaction pathways: (i) π–π stacking interactions, referring to non-covalent interactions between aromatic π-electron systems, (ii) hydrophobic interactions, and (iii) pore trapping (physical entrapment). π–π stacking interactions may contribute particularly to the adsorption of aromatic microplastic polymers such as polystyrene (PS). These mechanisms often act synergistically, contributing to enhanced adsorption efficiency and selectivity toward different types of microplastics. However, the relative contribution of individual interaction pathways may vary considerably depending on polymer chemistry, pore structure, surface functionality, and wastewater composition.

Graphene oxide (GO), a two-dimensional carbon nanomaterial, has attracted considerable attention due to its high surface area and abundant oxygen-containing functional groups [9,38]. In the case of microplastics removal, π–π stacking interactions between aromatic domains of GO and aromatic polymers such as polystyrene (PS) are frequently considered one of the dominant adsorption mechanisms as schematically illustrated in Figure 1, left [40]. In addition, π-electron-rich carbon surfaces may promote strong interfacial interactions with organic contaminants and polymeric particles [8,10]. At the same time, the adsorption performance of GO-based systems may be strongly influenced by sheet aggregation, oxidation degree, surface wettability, and dispersion stability in aqueous environments.

However, the adsorption behavior of GO toward microplastics remains strongly dependent on oxidation degree, sheet aggregation, and aqueous chemistry. While oxygen-containing functional groups may enhance surface reactivity and hydrogen-bonding interactions, excessive oxidation may simultaneously reduce hydrophobic graphitic domains and weaken π–π interactions with nonpolar polymers. Furthermore, aggregation of GO nanosheets in aqueous systems may significantly reduce accessible surface area and hinder transport within porous structures, leading to inconsistent adsorption performance across different studies [38,41].

Carbon nanotubes (CNTs), characterized by their one-dimensional graphitic structure and high aspect ratio, exhibit strong adsorption capability toward hydrophobic polymer particles. Hydrophobic interactions between CNT surfaces and nonpolar microplastics such as polyethylene (PE) and polypropylene (PP) are generally considered the dominant adsorption pathway (Figure 1, center) [42,43].

Despite their high adsorption capacity, CNT-based systems remain associated with several unresolved challenges. Strong hydrophobic interactions facilitate adsorption of nonpolar polymers; however, aggregation of CNTs in aqueous media may reduce active surface accessibility and limit adsorption efficiency. In addition, regeneration and recovery of dispersed CNTs from treated water remain difficult under practical operating conditions. Concerns related to potential nanotoxicity and environmental persistence of free CNTs further complicate their large-scale implementation in wastewater treatment systems [42,43,44].

Activated carbon remains one of the most widely used adsorbents in water treatment due to its highly developed porous structure and surface heterogeneity [39,45]. Pore trapping and surface adsorption, as illustrated in Figure 1, right, are considered the primary mechanisms governing microplastics immobilization within hierarchical pore networks. These mechanisms are particularly effective for irregularly shaped particles and fibrous microplastics.

Nevertheless, the adsorption performance of activated carbon may vary substantially depending on pore size distribution, activation method, and surface chemistry. Several studies have suggested that internal micropores may not be fully accessible to larger microplastic particles, particularly under dynamic flow conditions. Moreover, non-selective adsorption of dissolved organic matter and coexisting pollutants may accelerate pore saturation and reduce long-term adsorption efficiency in complex wastewater matrices [39,45].

Biochar, a carbon-rich material derived from biomass pyrolysis, has emerged as a sustainable and cost-effective alternative with tunable surface functionality and porous structure [42,46]. Depending on feedstock composition and pyrolysis conditions, biochar may exhibit hydrophobic domains, oxygen-containing functional groups, and hierarchical porosity capable of contributing to microplastics capture through hydrophobic attraction and pore trapping mechanisms.

However, one of the major challenges associated with biochar-based systems is the strong variability in physicochemical properties resulting from differences in biomass source, pyrolysis temperature, activation conditions, and post-treatment procedures. This variability complicates reproducibility and hinders direct comparison of adsorption performance among reported studies. In addition, the dominant adsorption mechanism in biochar systems is not yet fully resolved, as some studies emphasize hydrophobic attraction, whereas others suggest pore confinement and surface heterogeneity as the primary controlling factors [42,46].

Beyond conventional carbon materials, emerging engineered porous carbons and multifunctional nanocomposites have further expanded the design space for microplastics remediation [39,42]. Nevertheless, despite their promising adsorption performance, standalone carbon-based materials still exhibit important limitations, including aggregation in aqueous environments, reduced accessibility of active sites, limited regeneration efficiency, and insufficient structural stability under dynamic flow conditions [37,39].

Collectively, these limitations indicate that individual carbon materials may not simultaneously provide sufficient adsorption efficiency, operational stability, and long-term reusability under practical wastewater environments. Consequently, increasing attention has shifted toward hybrid material strategies capable of integrating structural robustness with multifunctional adsorption mechanisms. In particular, incorporation of carbon nanomaterials into biopolymer matrices such as cellulose has emerged as a promising approach to improve dispersion stability, stabilize active adsorption domains, and enhance structural integrity during repeated operation cycles.

Such carbon–cellulose composite systems enable synergistic interactions between structural scaffolds and adsorption-active carbon domains, offering improved performance for microplastics removal, as will be discussed in the following section.

To comparatively evaluate the advantages and limitations of cellulose-based materials, carbon-based adsorbents, and hybrid carbon–cellulose systems, Table 1 summarizes their dominant removal mechanisms, structural characteristics, and scalability potential. The comparison highlights the importance of hybrid material design for combining adsorption efficiency, structural stability, and practical applicability in wastewater treatment systems.

## 6. Carbon–Cellulose Composite Materials for Microplastics Removal from Wastewater

Carbon–cellulose composite materials have emerged as an advanced class of hybrid adsorbents that integrate the structural advantages of cellulose with the high adsorption capacity of carbon-based materials [47,48,49,50]. The combination of these components enables the design of multifunctional porous architectures with enhanced mechanical stability, tunable surface chemistry, and improved affinity toward microplastic particles [24,48,49,50].

Compared with pristine cellulose or standalone carbon materials, carbon–cellulose composites exhibit pronounced synergistic effects arising from interfacial interactions between cellulose hydroxyl groups and carbon nanostructures. These interactions promote improved dispersion of carbon phases, increased accessible surface area, and enhanced adsorption performance [48,49,50,51]. Hybrid systems are particularly attractive because they combine hydrophilic cellulose frameworks with hydrophobic carbon domains, enabling simultaneous interaction with a wide spectrum of polymer types [33].

Nevertheless, despite the generally improved adsorption performance of hybrid systems, the exact contribution of each component remains insufficiently understood. In many studies, enhanced adsorption efficiency is attributed broadly to “synergistic effects” without clearly distinguishing between the roles of pore trapping, hydrophobic interactions, π–π stacking, and surface adsorption. This lack of mechanistic clarification complicates direct comparison between reported systems and limits the establishment of universal structure–performance relationships.

### 6.1. Types of Carbon–Cellulose Composites

**Figure 2:**
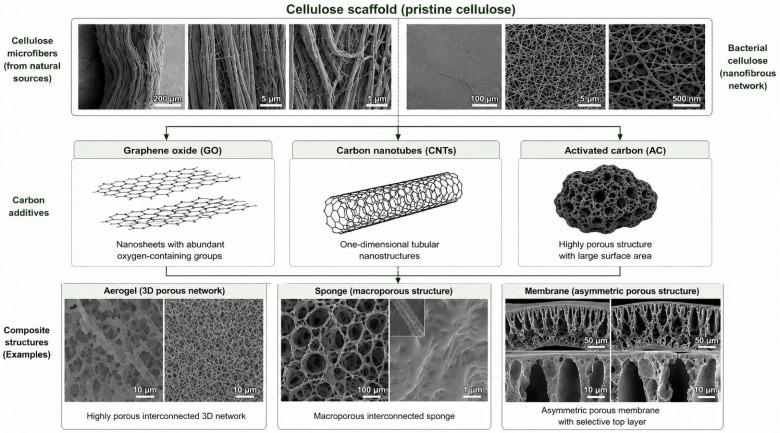
Schematic illustration of cellulose-based and carbon–cellulose hybrid porous architectures incorporating graphene oxide (GO), carbon nanotubes (CNTs), and activated carbon (AC) for microplastics adsorption and separation applications. Representative porous architectures and scaffold morphologies were adapted and redrawn based on Refs. [55,56,57,58] under Creative Commons licenses.

Carbon–cellulose composites can be broadly classified according to the type of carbon component and structural configuration, including cellulose–graphene oxide (GO) composites, cellulose–carbon nanotube (CNT) systems, cellulose–biochar hybrids, and cellulose–activated carbon materials [47,49,52]. Each class exhibits distinct interfacial properties, pore architectures, and adsorption behavior. As schematically illustrated in Figure 2, these composites are commonly fabricated as hierarchical porous structures in which carbon nanomaterials are distributed within a three-dimensional cellulose matrix. Such architectures promote efficient exposure of active adsorption sites while maintaining structural integrity.

However, the degree of carbon dispersion within cellulose matrices remains highly dependent on fabrication strategy and interfacial compatibility. Several studies have reported that excessive carbon loading may induce aggregation of graphene sheets or CNT bundles, resulting in pore blockage, reduced surface accessibility, and deterioration of mechanical stability [52,53,54]. Conversely, insufficient incorporation of carbon phases may limit hydrophobic interactions and reduce adsorption affinity toward nonpolar microplastics. Therefore, optimization of carbon content remains a major challenge in the rational design of hybrid adsorbents.

### 6.2. Synthesis Strategies

Various fabrication strategies have been developed to construct carbon–cellulose composites, including freeze-drying, in situ self-assembly, solution blending, vacuum filtration, hydrothermal synthesis, and chemical crosslinking [48,50,59]. Freeze-drying techniques are widely employed to fabricate ultralight aerogels with interconnected porous networks capable of trapping microplastic particles across broad size ranges [53]. In addition, in situ assembly approaches improve dispersion of carbon nanomaterials within cellulose matrices, thereby enhancing accessibility of adsorption-active domains [54].

Despite the diversity of synthesis approaches, methodological inconsistencies among studies remain substantial. Variations in drying conditions, solvent systems, crosslinking chemistry, carbon loading, and post-treatment procedures may significantly alter pore morphology and adsorption behavior. Consequently, adsorption efficiencies reported in the literature are often difficult to compare directly, even for apparently similar composite systems. Furthermore, many synthesis strategies remain difficult to scale up due to high energy consumption, prolonged processing time, or poor reproducibility of hierarchical pore structures.

### 6.3. Structure–Function Relationships and Adsorption Mechanisms

The performance of carbon–cellulose composites is primarily governed by their hierarchical structure and interfacial chemistry. Cellulose provides a three-dimensional scaffold enriched with hydroxyl functional groups, while carbon nanomaterials introduce hydrophobic domains and π-conjugated surfaces. This dual functionality enables multiple adsorption mechanisms to operate simultaneously. Hydrophobic interactions facilitate capture of nonpolar polymers such as polyethylene (PE) and polypropylene (PP) [43], whereas π–π stacking interactions contribute to adsorption of aromatic microplastics such as polystyrene (PS) [40]. Hydrogen bonding may additionally promote interaction with oxidized or aged microplastics, while interconnected porous networks facilitate capture of particles with diverse morphologies [51,54,59].

Although these interaction mechanisms are frequently discussed as complementary, their relative contribution remains debated. Some studies suggest that adsorption is primarily governed by hydrophobic and π–π interactions occurring on carbon surfaces, whereas others emphasize the dominant role of physical entrapment within hierarchical cellulose networks. In several studies, adsorption mechanisms are inferred indirectly from removal efficiency data without direct spectroscopic or surface characterization evidence. Consequently, mechanistic interpretation remains uncertain. This issue becomes particularly important for environmentally weathered microplastics, whose surface chemistry may differ substantially from pristine polymers.

The synergistic combination of these mechanisms generally enhances removal efficiency compared with single-component adsorbents, highlighting the importance of rational hybrid material design [48,50].

### 6.4. Adsorption Performance

Recent studies have demonstrated that carbon–cellulose composites exhibit high removal efficiencies across broad ranges of microplastic sizes and morphologies [52,59]. GO–cellulose aerogels and CNT–cellulose sponges have shown promising adsorption performance under optimized laboratory conditions due to their high surface area and interconnected porous structure [52,53]. However, adsorption efficiency strongly depends on critical design parameters, including carbon loading, pore size distribution, surface functionalization, and interfacial compatibility.

Reported adsorption performances vary substantially among studies because of differences in experimental conditions, including microplastic concentration, particle size distribution, adsorption time, water chemistry, and analytical quantification methods. In many cases, adsorption experiments are conducted using idealized laboratory suspensions containing single polymer types, which may not accurately reflect the complexity of real wastewater systems. Consequently, promising adsorption/removal performance under laboratory conditions may not necessarily translate into comparable performance under realistic environmental conditions.

Excessive carbon content may lead to aggregation and pore blockage, reducing adsorption efficiency, whereas insufficient carbon loading may limit hydrophobic interactions and adsorption capacity [54].

### 6.5. Reusability and Practical Considerations

An important advantage of carbon–cellulose composites is their potential reusability and operational stability. Many systems can be regenerated through mechanical compression, solvent washing, or mild thermal treatment while maintaining satisfactory adsorption performance over multiple cycles [53,59,60].

Nevertheless, long-term operational stability under operational wastewater systems remains insufficiently investigated. Fouling caused by dissolved organic matter, inorganic salts, and biofilm accumulation may progressively block adsorption sites and reduce pore accessibility during repeated operation. In addition, repeated adsorption–desorption processes may induce structural deformation of porous aerogels and sponges, leading to gradual deterioration of adsorption performance.

Another important limitation concerns scalability and process integration. Although laboratory-scale composites often demonstrate high adsorption efficiency under controlled experimental conditions, industrial implementation remains challenging due to material cost, reproducibility issues, regeneration complexity, and difficulties associated with continuous-flow operation. Therefore, future research should focus not only on maximizing adsorption capacity but also on improving long-term durability, economic feasibility, and practical applicability under complex wastewater treatment conditions.

As summarized in Table 2, carbon–cellulose composites can achieve promising adsorption/removal performance under optimized conditions, demonstrating strong potential as sustainable and potentially high-performance adsorbents for microplastics remediation [61].

## 7. Mechanisms of Microplastics Removal by Carbon–Cellulose Composites

The removal of microplastics by carbon–cellulose composite materials is governed by a combination of physical, chemical, and interfacial interactions. Unlike conventional adsorbents, which typically rely on a single dominant mechanism, hybrid carbon–cellulose systems exhibit multi-mechanistic adsorption behavior due to their heterogeneous surface chemistry, amphiphilic interfacial domains, and hierarchical porous structures [63,64,65,66,67,68,69,70,71,72].

The coexistence of cellulose-based porous scaffolds and carbon-derived active domains enables simultaneous contributions from physical entrapment, hydrophobic interactions, π–π stacking, hydrogen bonding, and electrostatic forces [24,68,72]. The synergistic interplay of these mechanisms significantly enhances adsorption efficiency and selectivity toward microplastics.

Although these mechanisms are frequently discussed collectively in the literature, adsorption, filtration, pore trapping, and physical entrapment represent distinct but interrelated processes. Adsorption generally refers to surface-mediated interactions occurring at active interfacial domains, including hydrophobic attraction, π–π stacking, hydrogen bonding, and electrostatic interactions. In contrast, filtration and size exclusion mainly involve mechanical retention of particles within interconnected porous structures. Physical entrapment and pore trapping represent intermediate processes in which microplastic particles become immobilized within hierarchical pore networks through combined steric confinement and interfacial interactions. In practical hybrid systems, these mechanisms often operate simultaneously and are difficult to distinguish experimentally.

However, despite the broad mechanistic framework proposed in the literature, the relative contribution of individual interaction pathways remains poorly understood. In many studies, adsorption mechanisms are inferred indirectly from removal efficiency data without direct spectroscopic, surface, or thermodynamic evidence. Consequently, distinguishing between surface adsorption, pore confinement, and interfacial trapping mechanisms remains challenging, particularly in complex hybrid systems containing multiple active domains.

### 7.1. Physical Entrapment, Filtration, and Size Exclusion

**Figure 3:**
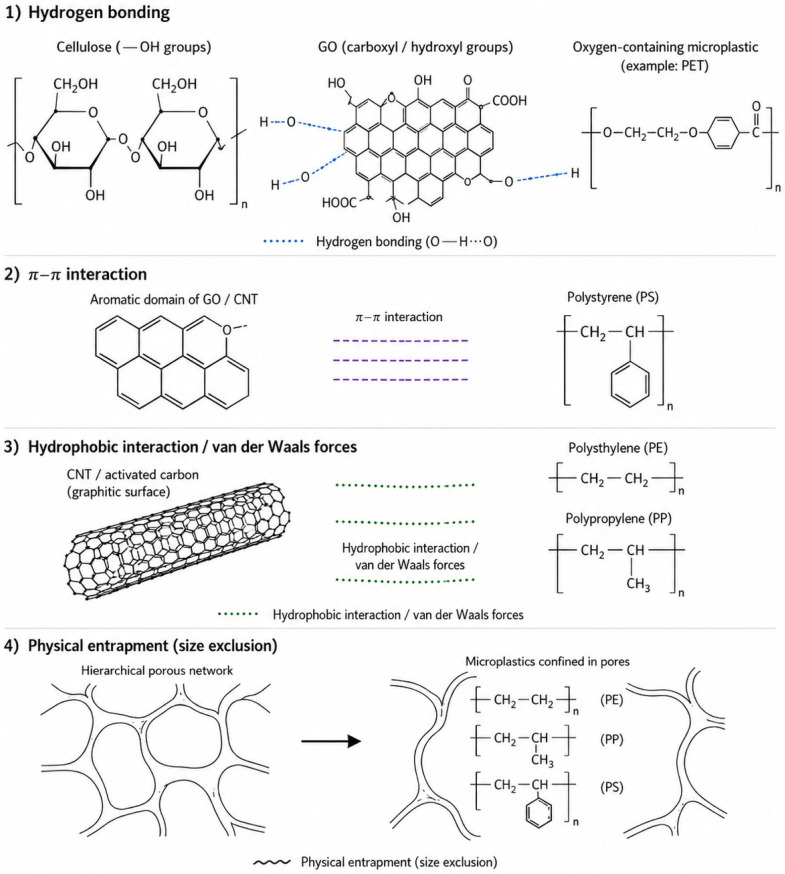
Conceptual distinction between adsorption, pore trapping, physical entrapment, and interfacial interaction mechanisms in carbon–cellulose composite systems for microplastics removal.

Physical entrapment is considered one of the dominant mechanisms governing microplastics capture in carbon–cellulose composites. In this process, microplastic particles are immobilized within interconnected porous networks of cellulose-based aerogels, sponges, or hierarchical hybrid structures [64,65,66,67]. As schematically illustrated in Figure 3, the coexistence of micro-, meso-, and macroporous domains enables efficient size exclusion and mechanical trapping of particles with diverse morphologies and dimensions. This mechanism is particularly important for fibrous and irregularly shaped microplastics, which may become mechanically interlocked within three-dimensional porous architectures.

Pore connectivity, tortuosity, and surface wettability strongly influence transport-limited capture behavior and retention efficiency [39,65].

Nevertheless, the actual role of physical entrapment remains difficult to quantify independently from surface adsorption effects. In highly porous aerogel systems, apparent adsorption enhancement may partially result from mechanical filtration and restricted particle mobility rather than true surface-mediated adsorption. Furthermore, variations in pore size distribution and particle dimensions among different studies complicate direct comparison of trapping efficiency.

### 7.2. Hydrophobic Interactions

Hydrophobic interactions represent a key mechanism governing adsorption of nonpolar polymers such as polyethylene (PE) and polypropylene (PP). Carbon domains within the composite—including graphitic surfaces, reduced graphene oxide regions, carbon nanotubes, and aromatic biochar structures—provide hydrophobic adsorption-active sites capable of preferential interaction with polymer surfaces [68,69,70,71].

The amphiphilic nature of carbon–cellulose composites, arising from the coexistence of hydrophilic cellulose and hydrophobic carbon phases, promotes interfacial adhesion and facilitates selective adsorption of polymer particles in aqueous systems [43]. The strength of hydrophobic interactions may additionally depend on polymer crystallinity, environmental aging, and solution chemistry.

However, the dominance of hydrophobic interactions remains debated, particularly for environmentally aged microplastics. Surface oxidation and weathering processes may introduce polar oxygen-containing groups onto polymer surfaces, thereby reducing hydrophobicity and altering adsorption behavior. Consequently, adsorption mechanisms observed for pristine laboratory-prepared microplastics may not accurately represent interactions occurring under realistic environmental conditions.

### 7.3. π–π Stacking Interactions

For aromatic microplastics such as polystyrene (PS), π–π stacking interactions between π-electron systems of carbon materials and aromatic polymer structures play an important role in adsorption performance [72,73,74]. This mechanism is particularly pronounced in graphene- and CNT-based composites, where extended conjugated carbon surfaces facilitate strong non-covalent interactions. Spectroscopic analyses have demonstrated that π–π interactions contribute significantly to the adsorption affinity of carbon-containing hybrid materials toward aromatic polymers [40].

Nevertheless, several studies suggest that the contribution of π–π stacking interactions may sometimes be overestimated due to the simultaneous occurrence of hydrophobic adsorption and pore confinement effects. In addition, aggregation of graphene sheets or CNT bundles may reduce accessibility of π-conjugated domains, thereby limiting effective interaction with aromatic microplastics. These competing effects remain insufficiently investigated in current adsorption studies.

### 7.4. Hydrogen Bonding and Surface Functional Interaction

Environmental aging of microplastics frequently leads to formation of oxygen-containing functional groups such as hydroxyl (–OH), carbonyl (–C=O), and carboxyl (–COOH) groups. These functionalities enable hydrogen bonding interactions with hydroxyl groups present in cellulose or oxidized carbon surfaces [69,75]. Such interactions may enhance interfacial adhesion and improve retention efficiency, particularly for aged microplastics exhibiting increased surface polarity [33].

This mechanism becomes especially important in real wastewater environments, where plastic particles undergo continuous physicochemical and oxidative transformation.

However, hydrogen bonding interactions are highly sensitive to solution chemistry, pH, ionic strength, and competitive adsorption effects. In complex wastewater matrices containing dissolved organic matter and coexisting contaminants, hydrogen-bonding sites may become partially blocked or screened, thereby reducing adsorption efficiency. These environmental effects are often neglected in simplified laboratory adsorption experiments.

### 7.5. Electrostatic Interactions

Electrostatic interactions also contribute to the overall adsorption process. Depending on pH and ionic strength, both cellulose and carbon surfaces may acquire positive or negative charges, enabling electrostatic attraction or repulsion with charged or partially oxidized microplastic particles [76,77,78]. Although electrostatic forces are generally weaker than hydrophobic or π–π interactions, they may influence adsorption behavior in wastewater matrices containing dissolved salts, natural organic matter, and competing contaminants.

The contribution of electrostatic interactions remains particularly difficult to predict because surface charge characteristics of both adsorbents and microplastics may vary dynamically depending on environmental aging, oxidation degree, and water chemistry. Furthermore, contradictory adsorption trends have been reported under different pH and salinity conditions, indicating that electrostatic effects may not operate independently but rather in combination with other interfacial mechanisms.

### 7.6. Synergistic Mechanisms in Hybrid Systems

The most distinctive feature of carbon–cellulose composites is the synergistic integration of multiple adsorption mechanisms within a single material system [63,68,72,77,78]. Cellulose provides a porous structural framework facilitating mass transport and physical entrapment, whereas carbon materials introduce adsorption-active interfacial domains responsible for hydrophobic interactions, π–π stacking, and surface-mediated adsorption.

The simultaneous contribution of multiple interaction pathways generally enhances adsorption capacity, adsorption kinetics, selectivity toward diverse microplastic types, and structural stability during repeated operation cycles [68,72].

Despite these advantages, mechanistic interpretation of synergistic effects remains challenging because multiple interaction pathways often occur simultaneously and cannot be easily isolated experimentally. In many cases, improved adsorption performance is attributed broadly to “synergistic behavior” without quantitative evaluation of individual mechanistic contributions. Therefore, future studies should focus on advanced spectroscopic characterization, adsorption thermodynamics, and in situ interfacial analyses to better elucidate structure–mechanism relationships in carbon–cellulose composite systems.

Overall, adsorption in carbon–cellulose composites should be considered as a coupled multiphase process involving simultaneous interfacial adsorption, porous confinement, and transport-limited retention rather than a single dominant mechanism.

## 8. Challenges and Future Research Directions

Despite significant progress in the development of carbon–cellulose composite materials for microplastics removal, several critical challenges still limit their large-scale implementation in real wastewater treatment systems [79,80,81,82]. These challenges are associated with methodological inconsistencies, material stability, process scalability, and insufficient validation under realistic environmental conditions.

### 8.1. Limitations of Current Materials

One of the major limitations is the lack of standardized methodologies for evaluating microplastics removal performance. Reported studies vary significantly in terms of particle size distribution, polymer composition, experimental conditions, and analytical techniques, making cross-comparison of adsorption efficiencies difficult and often unreliable [79,80]. As a result, adsorption performances reported in the literature may not always reflect intrinsic material superiority, but rather differences in experimental design and analytical methodology. Another critical issue is the discrepancy between laboratory-scale experiments and real wastewater systems. In real wastewater matrices containing dissolved organic matter, inorganic salts, surfactants, suspended solids, and co-existing pollutants, competitive adsorption, surface fouling, and blockage of active adsorption sites may significantly reduce removal efficiency and mass transfer accessibility within porous composite structures [81,82]. As emphasized in recent studies [24], these effects can significantly decrease removal efficiency compared to idealized laboratory conditions. In addition, suspended solids and surfactant-rich wastewater matrices may alter interfacial wettability and promote aggregation of carbon nanomaterials, thereby reducing adsorption accessibility and transport efficiency within porous composite networks. These effects are rarely considered in simplified laboratory adsorption studies.

Fouling and clogging of porous structures remain major operational challenges. In cellulose-based aerogels and sponges, prolonged exposure to wastewater may result in pore blockage, structural collapse, and decreased adsorption capacity over time [83].

Carbon-based nanomaterials, while highly efficient, also present intrinsic limitations such as aggregation in aqueous media, limited dispersion stability, and challenges associated with recovery and reuse. In addition, potential environmental risks associated with the release of dissociated nanoscale carbon particles during long-term operation and regeneration processes must be carefully considered [43,84,85].

Another important concern involves the potential release of nanoscale carbon components during long-term operation and regeneration processes. Leaching of graphene fragments or carbon nanotubes into treated water may introduce secondary environmental risks, highlighting the importance of structural stabilization and nanomaterial immobilization strategies.

### 8.2. Stability and Regeneration Issues

Reusability is a key requirement for practical applications. Although many carbon–cellulose composites demonstrate promising regeneration through mechanical compression, solvent washing, or mild thermal treatment, adsorption efficiency often decreases after repeated cycles due to incomplete desorption and structural degradation [86]. In practical wastewater systems, repeated adsorption–desorption cycles may additionally accelerate pore blockage, structural deformation, and irreversible fouling due to accumulation of natural organic matter and biofilm growth on porous composite surfaces. However, the mechanisms governing long-term structural degradation and irreversible fouling remain insufficiently understood. Mechanical stability under dynamic flow conditions remains insufficiently investigated. Lightweight aerogels and porous sponges may undergo deformation or fragmentation during long-term operation, leading to reduced performance [87]. Furthermore, irreversible fouling caused by natural organic matter and biofilm formation can significantly reduce the number of effective reuse cycles [88].

### 8.3. Scale-Up and Engineering Challenges

From an engineering perspective, scalability remains one of the most significant barriers. Many fabrication techniques, including freeze-drying and laboratory-scale self-assembly, are energy-intensive, time-consuming, and difficult to translate into industrial-scale production [89]. In many cases, synthesis procedures optimized at laboratory scale may lose structural reproducibility and pore uniformity during large-scale fabrication. In addition, integration of carbon–cellulose composites into existing wastewater treatment infrastructure—such as filtration units, membrane systems, or packed-bed reactors—remains at an early stage of development [90]. Cost is another limiting factor, particularly when high-value nanomaterials such as graphene oxide and carbon nanotubes are used. The development of cost-effective alternatives is essential for practical implementation [91]. Furthermore, maintaining mechanical integrity under continuous-flow operation remains a critical engineering challenge, particularly for ultralight aerogels and highly porous sponge-like structures susceptible to compression, deformation, and structural collapse during prolonged use.

### 8.4. Future Research Directions

Future progress in this field will require not only improvement of adsorption capacity, but also deeper mechanistic understanding, realistic wastewater validation, standardized evaluation protocols, and scalable engineering solutions. To address these limitations, several promising research directions can be identified:

(1) Rational material design. Future work should focus on structure–property relationships, including optimization of pore size distribution, interfacial chemistry, and carbon loading to maximize adsorption efficiency and selectivity [92].
(2) Sustainable and low-cost carbon sources. The use of biomass-derived carbon materials (e.g., biochar) and waste-derived carbon nanostructures offers a promising pathway to reduce cost and improve environmental sustainability [93].
(3) Magnetic and multifunctional composites. Incorporation of magnetic nanoparticles (e.g., Fe_3_O_4_) enables efficient separation, improved recyclability, and potential application in dynamic treatment systems. Recent studies on superhydrophobic magnetic materials have demonstrated high efficiency and reusability in wastewater treatment processes [94].
(4) Real wastewater validation. Future studies must prioritize validation under real wastewater conditions rather than synthetic model systems to better assess material performance, stability, and long-term applicability [95].
(5) Mechanistic and modeling approaches. Advanced characterization techniques combined with computational modeling can provide deeper insights into adsorption kinetics, interfacial interactions, and degradation mechanisms [96].

### 8.5. Perspective

Carbon–cellulose composite materials represent a highly promising and sustainable platform for microplastics remediation. Their multifunctional nature, combining porous biopolymer scaffolds with high-performance carbon domains, enables efficient capture of diverse microplastic pollutants. Importantly, recent developments in superhydrophobic and magnetic nanocomposites highlight the potential of integrating advanced functionalities such as selective adsorption, recyclability, and easy separation [94,95]. In addition, studies on carbon-based hybrid materials derived from natural precursors demonstrate the feasibility of developing environmentally friendly and cost-effective solutions [96]. However, bridging the gap between laboratory research and real-world application requires interdisciplinary integration of materials science, environmental engineering, process modeling, and regulatory frameworks [97].

## 9. Conclusions

Microplastics contamination in wastewater represents a complex and persistent environmental issue that cannot be effectively addressed by conventional treatment technologies alone. Their small size, diverse morphology, and dynamic surface properties significantly limit removal efficiency and contribute to their continuous release into aquatic environments. In this review, carbon-based materials and cellulose-based materials have been critically analyzed as promising platforms for microplastics removal. Carbon materials offer high adsorption capacity and strong interfacial interactions through hydrophobic attraction and π–π stacking, while cellulose provides a sustainable, biodegradable scaffold with tunable surface functionality and hierarchical porosity. The integration of these two material classes into carbon–cellulose composites enables the development of multifunctional adsorbents with enhanced structural stability, improved dispersion of active components, and synergistic adsorption mechanisms. These hybrid systems combine physical entrapment, hydrophobic interactions, hydrogen bonding, electrostatic effects, and pore trapping mechanisms, resulting in improved removal efficiency across a wide range of microplastic types.

Despite these advantages, several critical challenges remain. The lack of standardized methodologies for microplastics detection and performance evaluation limits comparability between studies. In addition, significant variations in reported adsorption efficiencies arise from differences in polymer type, particle size distribution, wastewater composition, water chemistry, and experimental methodology. Material stability, fouling, regeneration efficiency, and scalability also require further investigation to enable practical implementation in real wastewater treatment systems. Moreover, the relative contribution of adsorption, filtration, physical entrapment, and interfacial interactions remains difficult to quantify in many hybrid systems, highlighting the need for more rigorous mechanistic investigations.

Future research should focus on the rational design of materials with optimized structure–property relationships, the development of cost-effective and sustainable carbon sources, and the incorporation of advanced functionalities such as magnetic separation and surface wettability control. In addition, validation under real wastewater conditions and integration into existing treatment processes are essential steps toward practical application. Particular attention should also be devoted to long-term operational stability, resistance to fouling, regeneration behavior, and the potential release of carbon nanomaterials during repeated use.

Overall, carbon–cellulose composite materials represent a highly promising and versatile platform for addressing the challenges of microplastics removal. Continued interdisciplinary research combining materials science, environmental engineering, and process design will be crucial for translating laboratory-scale innovations into scalable and sustainable wastewater treatment solutions. Rather than presenting carbon–cellulose composites as a fully established solution, this review highlights both the significant potential and the current limitations of these hybrid systems, while providing a focused critical perspective on future directions for realistic wastewater treatment applications. The present review additionally emphasizes the importance of integrating mechanistic understanding, standardized evaluation protocols, and practical engineering considerations in the future development of next-generation carbon–cellulose nanocomposite adsorbents for sustainable microplastics remediation.

In this context, the development of sustainable carbon–cellulose hybrid materials may contribute to global efforts toward achieving Sustainable Development Goal 6 (Clean Water and Sanitation) through improved removal of emerging contaminants from wastewater systems.

## Figures and Tables

**Table 1:** Comparative advantages, limitations, dominant removal mechanisms, and scalability potential of cellulose-, carbon-, and carbon–cellulose-based materials for microplastics remediation.

Material System	Main Advantages	Key Limitations	Dominant Removal Mechanism	Scalability Potential
Cellulose-based materials	Renewable, biodegradable, low toxicity, tunable porous structure, high hydrophilicity	Low affinity toward hydrophobic polymers, swelling, fouling, limited long-term stability	Physical entrapment, filtration, hydrogen bonding	High
Carbon-based materials	High specific surface area, strong adsorption affinity, hydrophobic domains, π-conjugated structures	Aggregation, difficult recovery, possible nanotoxicity, pore blockage, high cost	Hydrophobic interaction, π–π stacking, surface adsorption, pore trapping	Moderate
Carbon–cellulose composites	Synergistic adsorption behavior, improved dispersion stability, hierarchical porosity, multifunctional interfacial interactions, enhanced reusability	Complex fabrication, regeneration challenges, scale-up limitations, structural degradation during repeated cycles	Combined adsorption, hydrophobic interaction, π–π stacking, physical entrapment, pore confinement	Promising
Magnetic carbon–cellulose composites	Easy magnetic separation, improved recyclability, rapid recovery from aqueous systems	Nanoparticle leaching risk, synthesis complexity, stability under long-term operation	Magnetic-assisted adsorption, hydrophobic interaction, pore trapping	Moderate to high
Biochar–cellulose hybrid systems	Sustainable feedstocks, low cost, environmentally friendly production	Variable physicochemical properties, inconsistent adsorption performance	Hydrophobic interaction, pore filling, surface adsorption	High

**Table 2:** Comparative performance and practical limitations of representative carbon–cellulose composite systems for microplastics removal.

Composite System	Microplastic Type	Reported Performance	Dominant Mechanism	Main Limitation	Ref.
Wood-derived cellulose nanofibril aerogel	MPs	Reported efficient separation of broad-size MPs under laboratory conditions	Physical entrapment, pore trapping, filtration	Structural deformation and scale-up challenges	[58,59]
Magnetic carbon nanotubes	MPs	Effective removal observed in aqueous suspensions	Hydrophobic interaction, magnetic-assisted separation	CNT aggregation and secondary release risk	[44]
Biochar-based composite systems	Mixed MPs	Promising adsorption/capture performance	Pore filling, hydrophobic interaction	Strong dependence on feedstock and pyrolysis conditions	[46]
Cellulose/carbon composite systems	Various MPs/pollutants	Improved adsorption behavior under laboratory conditions	Surface adsorption, pore confinement, interfacial interaction	Difficult cross-study comparison due to varying test conditions	[16,62]
Cellulose nanofibril-loaded filter paper	MPs	High retention efficiency for multiscale particles	Filtration, physical retention	Sensitive to flow conditions and particle morphology	[17]
3D reduced graphene oxide	PS MPs	Effective adsorption of PS microplastics in laboratory studies	π–π interaction, hydrophobic adsorption	rGO aggregation reducing accessible surface area	[40]

## Data Availability

The data supporting this study are derived from publicly available sources cited within the article.

## Abbreviations

The following abbreviations are used in this manuscript:

MPs	Microplastics
WWTPs	Wastewater Treatment Plants
PE	Polyethylene
PP	Polypropylene
PS	Polystyrene
PET	Polyethylene Terephthalate
CNF	Cellulose Nanofibers
CNC	Cellulose Nanocrystals
GO	Graphene Oxide
rGO	Reduced Graphene Oxide
CNTs	Carbon Nanotubes
AC	Activated Carbon
BC	Biochar
π–π	Pi–Pi Interactions
SDGs	Sustainable Development Goals
